# Clinical Significance of Asthma Clusters by Longitudinal Analysis in Korean Asthma Cohort

**DOI:** 10.1371/journal.pone.0083540

**Published:** 2013-12-31

**Authors:** So Young Park, Seunghee Baek, Sujeong Kim, Sun-young Yoon, Hyouk-Soo Kwon, Yoon-Seok Chang, You Sook Cho, An-Soo Jang, Jung Won Park, Dong-Ho Nahm, Ho-Joo Yoon, Sang-Heon Cho, Young-Joo Cho, ByoungWhui Choi, Hee-Bom Moon, Tae-Bum Kim, for the COREA Study Group

**Affiliations:** 1 Division of Allergy and Clinical Immunology, Department of Internal Medicine, Asan Medical Center, University of Ulsan College of Medicine, Seoul, Korea; 2 Department of Clinical Epidemiology and Biostatistics, Asan Medical Center, Seoul, Korea; 3 Department of Internal Medicine, Seoul National University College of Medicine, Seoul, Korea; 4 Department of Internal Medicine, Soonchunhyang University Bucheon Hospital, Bucheon, Korea; 5 Department of Internal Medicine, College of Medicine, Yonsei University, Seoul, Korea; 6 Department of Internal Medicine, College of Medicine, Ajou University, Suwon, Korea; 7 Department of Internal Medicine, College of Medicine, Hanyang University, Seoul, Korea; 8 Department of Internal Medicine, College of Medicine, Ewha Womans University, Seoul, Korea; 9 Department of Internal Medicine, College of Medicine, Chung-Ang University, Seoul, Korea; Cincinnati Children's Hospital Medical center, United States of America

## Abstract

**Background:**

We have previously identified four distinct groups of asthma patients in Korean cohorts using cluster analysis: (A) smoking asthma, (B) severe obstructive asthma, (C) early-onset atopic asthma, and (D) late-onset mild asthma.

**Methods and Results:**

A longitudinal analysis of each cluster in a Korean adult asthma cohort was performed to investigate the clinical significance of asthma clusters over 12 months.

Cluster A showed relatively high asthma control test (ACT) scores but relatively low FEV_1_ scores, despite a high percentage of systemic corticosteroid use. Cluster B had the lowest mean FEV_1_, ACT, and the quality of life questionnaire for adult Korean asthmatics (QLQAKA) scores throughout the year, even though the percentage of systemic corticosteroid use was the highest among the four clusters. Cluster C was ranked second in terms of FEV_1_, with the second lowest percentage of systemic corticosteroid use, and showed a marked improvement in subjective symptoms over time. Cluster D consistently showed the highest FEV_1_, the lowest systemic corticosteroid use, and had high ACT and QLQAKA scores.

**Conclusion:**

Our asthma clusters had clinical significance with consistency among clusters over 12 months. These distinctive phenotypes may be useful in classifying asthma in real practice.

## Introduction

Asthma is a clinical syndrome of intermittent respiratory symptoms characterized by chronic airway inflammation, nonspecific bronchial hyperresponsiveness, and reversible airway obstruction [1]. In the past, asthma management depended on the severity of the disease, which is determined according to patient lung function [forced expiratory volume in 1 second (FEV_1_)], daytime and nocturnal symptoms, and the frequency of rescue bronchodilator use [2]. Recent treatment guidelines emphasize the asthma control status, a convenient approach that can easily be applied clinically [1]. However, both the asthma control and severity have demonstrated critical drawbacks by failing to reflect the heterogeneous nature of asthma, which is determined by individually distinct pathophysiological backgrounds [3]–[5]. The concept of ‘heterogeneity’ has been used on a number of occasions in an attempt to create a novel classification that integrates heterogeneous clinical characteristics with intrinsic disease severity, facilitating tailored treatment for asthma patients [5]–[8].

In our previous study [9], we used cluster analysis to identify four distinct groups of asthma in two large independent cohorts of Korean adult asthma patients: (A) smoking asthma, (B) severe obstructive asthma, (C) early-onset atopic asthma, and (D) late-onset mild asthma. Both cohorts were comprised of asthma patients at all levels of severity and had a reasonably large of number of participants [10].

Because the cluster analysis classifications in previous similar studies [5]–[8] lacked longitudinal evaluation of the population, it was difficult to confirm the clinical significance and to justify the real clinical relevance of the classifications. To overcome this limitation, our previous study revealed the change of FEV_1_ throughout a 12-month follow-up period in the four distinctive groups [10].

The purpose of our present study was to characterize and compare cluster-specific trends in clinical parameters of asthma control over 12 months of follow-up in a Korean asthma cohort.

## Methods

### Study population

This study was performed with 724 subjects from the Cohort for Reality and Evolution of Adult Asthma in Korea (COREA), who were the subjects of our previous cluster analysis [10]. As described in the previous report, patients, recruited by allergists or pulmonologists from 11 tertiary referral centers in Korea, were diagnosed with asthma by the presence of asthma symptoms and airway reversibility. The patients were seen at their hospitals regularly every 3 months and were managed uniformly according to the Global Initiative for Asthma (GINA) guidelines [1]. In every 3-month-visit, patients were supposed to check spirometry, report ACT scores and the investigator reviewed and recorded the medications taken in the previous 3 months. This included whether the patient had taken systemic corticosteroid for asthma exacerbation and whether asthma medication had been stepped up, down or maintained. Also, patients were asked to answer the questionnaire, QLQAKA, at cohort registration and every 1 year afterwards [11].

### Ethics

All study participants were fully informed of the study protocol and provided written, signed statements of informed consent. The protocol was approved by the Institutional Review Board and Ethics Committee of Asan Medical Center.

### Cluster analysis

Hierarchical cluster analysis using Ward's method was performed to estimate the number of likely clusters within the population and was followed by k-means cluster analysis as the principal clustering technique for grouping individuals into clusters [12]. Variables for modeling were selected based on their contribution to the characterization of the asthma phenotype, which were the FEV1, body mass index (BMI), age at onset, atopic status, smoking history, and history of hospital utilization due to exacerbation [10].

### Longitudinal outcome measures

We investigated the longitudinal trend of clinical status in terms of FEV_1_% predicted, asthma control test (ACT) score [13], percentage of systemic corticosteroid use (percentage of patients on a brief course of systemic corticosteroid for acute exacerbation at a given timepoint), and quality of life questionnaire for adult Korean asthmatics (QLQAKA) score [14] for each predefined cluster. Data at3^rd^, 6^th^, 9^th^ and 12^th^ months were retrieved from the cohort and were retrospectively analyzed.

QLQAKA is the Korean modified version of the Juniper asthma quality of life questionnaire (AQLQ) [15]. In the QLQAKA, answers to each question are scored on a 5-point scale, with a score of 1 representing greatest impairment and a score of 5 representing no impairment. Items are weighted equally and are reported as a mean score for each domain (activity limitations, emotions, symptoms, and exposure to environmental stimuli) along with the overall mean score [14].

### Statistical analysis

We used the linear mixed effects models [16] for the FEV_1_% predicted, the ACT and QLQAKA scores,and the generalized estimating equations (GEE) [17] for the percentage of systemic corticosteroid use in order to estimate the outcome values and test the differences among clusters [18]. Both statistical methods are used to account for within-individual correlations since the data for each patient were repeatedly measured and assumed to be correlated. The linear mixed effect model included cluster effect, time, and their interaction as fixed effects, and subject and time as random effects.The analyses were applied to the raw data with no imputation of missing data. To ensure the insensitivity of the results to missing data, analyses were also done after multiple imputations. Multiple imputations with a Markov chain Monte Carlo method were performed for each outcome using PROC MI in SAS, and 10 imputed data sets were then analyzed by using standard procedures for complete data. The results from the data sets were combined using PROC MIANALYZE in SAS. (See Supplementary Appendix) All statistical analyses were performed with SAS software (version 9.2; SAS Institute, Cary, NC, USA).

## Results

### FEV_1_

Because the patients whose FEV_1_ was only checked at baseline (with no follow-up data from the subsequent 12-month period) were excluded, the total number of subjects included in the analysis was 480. The distribution of the subjects at baseline for clusters A, B, C, and D was 57, 109, 159 and 155, respectively.

**Figure 1:**
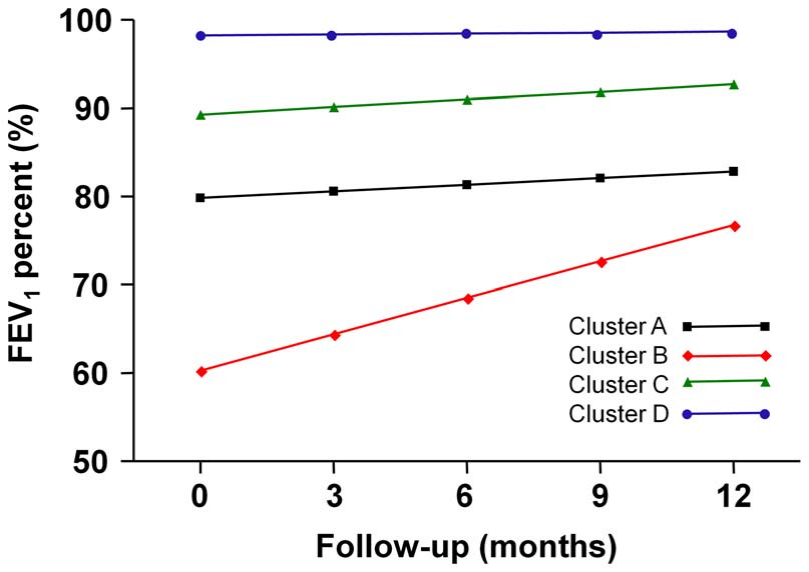
FEV_1_% predicted values during the 12-month follow-up period in each cluster (adapted from Kim TB et al., Eur Respir J, 2013;41:1308–1314.).

Although we previously published the change ofthe FEV_1_ during a 12-month period in four clusters [10], a more in-depth analysis has been performed here. The FEV_1_ is consistently lowest in cluster B (the severe obstructive asthma group) and highest in cluster D (the late-onset mild asthma group) (Figure 1 and Table 1). Cluster A, the smoking asthma group and cluster C, the early-onset atopic asthma group ranked between cluster B and D in the order of low FEV_1_. The differences between the four clusters throughout the 12-month follow-up period were statistically significant (*P*<0.0001) [10]. The improvement in the FEV_1_% predicted in cluster B over time was statistically significant (*P*<0.0001), whereas increases in the FEV_1_ in other clusters were not significant.

**Table 1:** FEV_1_% predicted valuesduring the 12-month follow-up period in each cluster.

	A		B		C		D	
Months	n	Pred. Mean (95% CI)	n	Pred. Mean (95% CI)	n	Pred. Mean (95% CI)	n	Pred. Mean (95% CI)
**0**	57	79.85 (76.70–83.00)	109	60.31 (58.02–62.60)	159	89.33 (87.46–91.22)	155	98.22 (96.30–100.15)
**3**	48	80.60 (77.53–83.68)	82	64.43 (62.19–66.66)	125	90.17 (88.33–92.02)	114	98.34 (96.47–100.22)
**6**	39	81.35 (78.10–84.60)	77	68.55 (66.19–70.91)	102	91.02 (89.06–92.98)	110	98.46 (96.48–100.44)
**9**	41	82.10 (78.46–85.74)	71	72.67 (70.02–75.31)	106	91.86 (89.66–94.07)	97	98.58 (96.36–100.80)
**12**	37	82.85 (78.67–87.03)	64	76.78 (73.73–79.84)	94	92.71 (90.17–95.25)	95	98.70 (96.15–101.25)

In order to check if improvement of FEV_1_ seen in cluster B represents loss of data from patients with a low baseline FEV_1_, we compared the mean values of last recorded FEV_1_ between patients who revisited the clinic and those who did not at each follow up point, however, we found no significant differences of the last recorded FEV_1_ at 3, 6, 9 months and interestingly, at 12 month, the mean value of last recorded FEV_1_ in the patients who did not revisit clinic was significantly higher than those who did.

### ACT score

**Figure 2:**
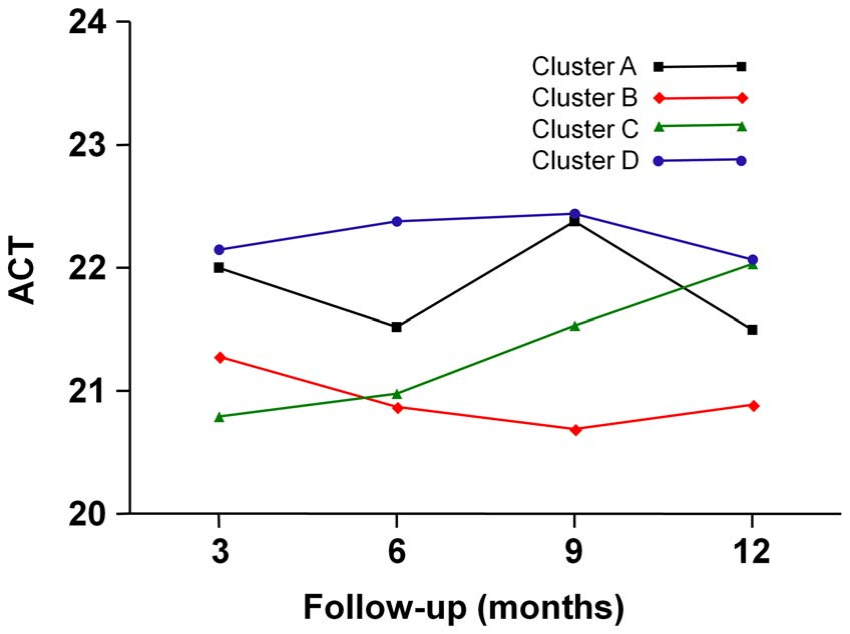
ACT scores during the 12-month follow-up period in each cluster.

The ACT score was measured from the 3^rd^ month (Figure 2 and Table 2). Patients who did not fill inthe ACT score were excluded from the study, leaving 459 patients. The distribution of patient numbers for clusters A, B, C, and D was 54, 102, 156 and 147, respectively.

**Table 2:** ACT scores during the 12-month follow-up period in each cluster.

	A		B		C		D	
Months	n	Pred. Mean (95% CI)	n	Pred. Mean (95% CI)	n	Pred. Mean (95% CI)	n	Pred. Mean (95% CI)
**3**	44	22.00 (21.05–22.96)	58	21.28 (20.49–22.07)	99	20.79 (20.18–21.41)	92	22.15 (21.51–22.78)
**6**	37	21.52 (20.44–22.61)	63	20.87 (20.05–21.69)	87	20.98 (20.30–21.67)	92	22.38 (21.70–23.06)
**9**	34	22.38 (21.35–23.41)	62	20.69 (19.93–21.45)	98	21.53 (20.92–22.13)	92	22.44 (21.82–23.07)
**12**	41	21.50 (20.53–22.46)	68	20.89 (20.15–21.63)	99	22.03 (21.42–22.64)	95	22.07 (21.45–22.69)

All mean ACT scores were greater than 20, even in the most severe group (cluster B). There was a tendency of the patients in cluster D to report higher ACT scores than patients in cluster A, B and C, with significant differencesfrom cluster B and C (*P* = 0.001 and 0.007, respectively). Patients in cluster B reported the lowest ACT scores throughout the whole follow-up period, with the exception of the 3^rd^month. Cluster A showed relatively high ACT scores compared with its low FEV_1_. A significant increase in ACT score was found in cluster C (*P* = 0.001).

### Percentage of systemic corticosteroid use

**Figure 3:**
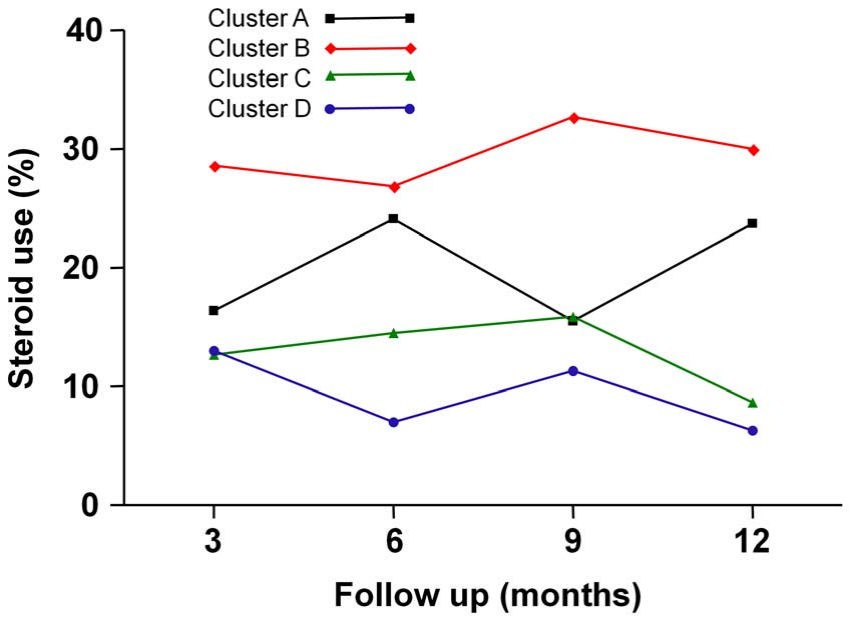
Percentage of use of systemic corticosteroids during the 12-month follow-up period in each cluster.

The number of patients used for longitudinal analysis of systemic corticosteroid use was 57, 109, 162, and 157 for clusters A, B, C and D, respectively. Patients for whom we had no information on systemic corticosteroid use were excluded from analysis. Patients in cluster B tended to morefrequently use systemic corticosteroids than patients in other clusters (Figure 3 and Table 3). In particular, the use of systemic corticosteroids in cluster B was significantly higher than in clusters C and D over the entire period and at each visit (*P* = 0.0005 and *P*<0.0001, respectively).The percentage of systemic corticosteroid use in each cluster at each visit displayed a fluctuating pattern.

**Table 3:** Percentage of use of systemic corticosteroids during the 12-month follow-up period in each cluster.

	A		B		C		D	
Months	n	Pred. Mean (95% CI)	n	Pred. Mean (95% CI)	n	Pred. Mean (95% CI)	n	Pred. Mean (95% CI)
**3**	52	0.164 (0.086–0.292)	84	0.286 (0.204–0.386)	130	0.127 (0.081–0.194)	124	0.130 (0.081–0.201)
**6**	42	0.241 (0.140–0.382)	79	0.269 (0.187–0.372)	108	0.145 (0.091–0.222)	115	0.070 (0.036–0.132)
**9**	43	0.155 (0.075–0.295)	74	0.327 (0.237–0.431)	112	0.159 (0.101–0.238)	105	0.113 (0.068–0.183)
**12**	41	0.237 (0.136–0.383)	68	0.300 (0.210–0.408)	101	0.087 (0.045–0.161)	102	0.063 (0.029–0.129)

### QLQAKA score

**Figure 4:**
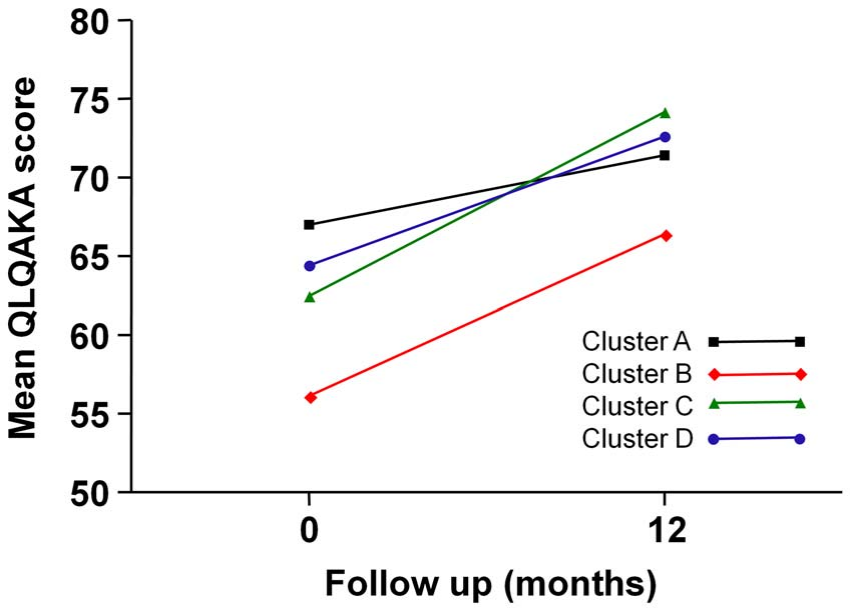
QLQAKA scores during the 12-month follow-up period in each cluster.

We measured QLQAKA scores twice, at baseline and at the 12^th^ month. Lower QLQAKA scores were consistently seen in cluster B than in other clusters (Figure 4 and Table 4). The differences between cluster B and the other clusters were statistically significant at baseline and the 12^th^ month, though no significant differences between cluster A and D were seen at the 12^th^ month. The scores from baseline to the 12^th^ month significantly increased in clusters B, C, and D (*P* = 0.001, *P*<0.0001, and *P*<0.003, respectively).

**Table 4:** QLQAKA scores during the 12-month follow-up period in each cluster.

	A		B		C		D	
Months	n	Pred. Mean (95% CI)	n	Pred. Mean (95% CI)	n	Pred. Mean (95% CI)	n	Pred. Mean (95% CI)
**0**	14	67.02 (60.79–73.26)	33	56.14 (52.07–60.20)	42	62.45 (58.85–66.04)	32	64.41 (60.30–68.52)
**12**	17	71.44 (65.77–77.12)	28	66.42 (62.02–70.81)	45	74.20 (70.73–77.68)	39	72.64 (68.90–76.38)

### Analyses after multiple imputations

In order to ensure the insensitivity of the results to missing data, we repeated the analyses after multiple imputations (10 times). Although there were slight differences in the absolute numbers in the results, the trends were almost identical to the original outcomes for the FEV_1_, ACT, and QLQAKA scores. For the percentage of corticosteroid use, the overall trend was similar, though not identical (Figures S1, S2, S3, S4 and Tables S1, S2, S3, S4).

## Discussion

As a follow-up to our previous report, we analyzed longitudinal outcomesin our Korean cohort to verify the validity and clinical significance of our clustering. We proposed a new differentiated classification model with easily accessible clinical variables on the basis of our previous asthma clustering [10]. However, we needed to confirm the usefulness and clinical application of our clinical phenotype classifications by observing their longitudinal changes, which has not yet been shown in other studies from Europe and the USA [5], [6]. To our knowledge, this is the first assessment of the longitudinal outcomes of asthma phenotypes by cluster analysis.

We analyzed the four variables that we considered to be the most clinically important.The FEV_1_ is an objective measure that reflects the severity and control status of asthma as well as the degree of airway obstruction. The use of systemic corticosteroids is also an important factor defining asthma control status because it is prescribed when patients are considered to be uncontrolled or only partly controlled [1]. In addition, we evaluated patterns in the ACT and QLQAKA scores as subjective means to assess asthma control status.

Because we observed changes in the values of each variable over time, we were able to outline the clinical course of each individual cluster. Cluster A (the smoking asthma group) had the second lowest mean FEV_1_% predicted after cluster B (the severe obstructive asthma group). Despite substantial airway obstruction, the ACT scoresof cluster A were, after those of cluster D (the late-onset mild asthma group), the second highest. From this speculation, we could hypothesize that the smoking patients might be underestimating the seriousness of their asthma symptoms, believing the symptoms to be related with smoking, or they might have blunted perception of airway obstruction.In fact, it is supported by Kleis et al who have shown smoking asthmatics have reduced dyspnea perception during a nonspecific provocative challenge with methacholine [19]. Moreover, patients in cluster A show high degree of blood eosinophil count compared with other clusters (Table S5) which is a supporting evidence for dissociation between symptom and severe exacerbations in eosinophilic asthma with high degree of airway inflammation as asserted by some researchers [20]. All patients in cluster A were diagnosed as asthma, not COPD, by allergists and pulmonologists which was assured with methacholine bronchial provocation test. Another point that was observed from longitudinal analysis was that, even with regular inhaled corticosteroid treatment, a statistically significant improvement in the FEV_1_ was not evident. This may be due to differences in the underlying pathophysiology of smoking asthma, attributable to the deleterious effect of cigarette smoking on asthma [21]. Unfortunately, this has limited clinical value since there is no data available regarding whether subjects have continued, reduced or quit smoking during the follow-up period.

The patients in cluster B showed the lowest mean FEV_1_% predicted, the highest percentage of systemic corticosteroid use, and the lowest ACT and QLQAKA scores throughout the 12-month follow-up period. Considering that the mean FEV_1_ consistently increased from 60.31% at baseline to 76.78% at the 12^th^ month with a marked improvement in QLQAKA scores, the patients in this cluster appear to respond to asthma treatment very well.In fact, they showed the largest percent increase in the FEV_1_after using short-acting bronchodilators in our previous study [10]. This implicates that we can expect increase in the FEV_1_ in the patients of this cluster if they are treated with the optimal asthma medications. However, the final mean FEV_1_% predicted of the patients in this cluster did not reach 80%, implying that they may be included in the severe refractory asthma group. Therefore, optimal management of these patients requires close monitoring from physicians. Furthermore, researchers should use this group to study the pathogenesis of severe refractory asthma.

Cluster C (the early-onset atopic asthma group) was, after cluster D, ranked second in terms of mean FEV_1_% predicted and had the second lowest percentage of systemic corticosteroid use. The mean FEV_1_ slightly, but not significantly, increased during the 12-month follow-up period.Interestingly, the ACT scores steadily increased from the 3^rd^ month to the last follow-up, meaning that the patients with early-onset atopic asthma may have a benign disease course with marked improvement of subjective symptoms. In contrast, Moore et al. [6] demonstrated an atopic subgroup of patients presenting with a severe course of disease. However, there were no severe asthma patients with a low baseline FEV_1_ and a poor prognosis in this cluster in our study.

The patients in cluster D consistently showed the highest FEV_1_% predicted, the lowest percentage of systemic corticosteroid use, and high ACT and QLQAKA scores. The longitudinal outcome analysis demonstrated its benign course throughout the 12-month period, which is comparable to the ‘benign asthma’ group in the United Kingdom study [5]. One unique finding was that this mild asthma group of patients was comprised of those with a later onset of disease andhad a female predominance. A common feature of female predominant, older subgroups from other studies was that the patients were obese and non-eosinophilic [5], [6]. However, our patients were not obese compared to patients from other clusters and they did not show significantly different total serum IgE levels or blood eosinophil counts [10]. This is indicative of the distinctness of the Korean asthma population, consisting of a subgroup of older, female predominant, asthma patients showing a benign course of disease.

Another interesting finding from this study was that all ACT scores were higher than 20. Usually, in practice we assess patients to be fairly well controlled if the scores are equal to or higher than 20 [22]. Most patients recruited in the cohort appeared to be well managed by attending physicians, because most showed reasonably good lung function during the follow-up period. However, because even those patients of cluster B with relatively poor lung function reported ACT scores greater than 20, physicians should recognize the fact that patients may underestimate their status when scoring ACT.

We didn't provide information on medication use in this article. The patients were managed by the GINA guidelines, and accordingly the medications were stepped up, down or maintained based on their asthma control status. Therefore, the level of controller medication may differ depending on the asthma severity even for patients in the same cluster, and it may also change in one patient at different follow up points. We thought that change in medication use over time does not necessarily charge bias in the overall picture of the clinical course, since all patients were managed under one big principle. Indeed, when we analyzed the patients' self-reported compliance to their prescriptionsby a visual-analogue scale at every 3-month-visit, we found that all mean scores for every cluster was above 80%, which is fairly good. Therefore, we believe that difference in medications did not influence our results since most patients were compliant to their prescriptions that adhered to the GINA guidelines.

A major limitation of the present study is that we could not complete the 12-month database with all subjects who participated in the initial cluster analysis. There was loss to follow-up and missing data for all variables. In order to overcome this problem, we used the linear mixed effect model and GEE, which offer a general framework from which to develop longitudinal analyses under the missing at random (MAR) assumption [23], so these methods are more robust to potential bias from missing data than LOCF(Last Observation Carried Forward). These methods provided us with unbiased estimates of cluster and time effects assuming that the data loss occurred randomly. Simultaneously, they defined the longitudinal trend of each cluster and make comparisons between them [18]. To confirm the sensitivity of the missing data, we performed multiple(10) imputationsto fill in the missing variables and combined the 10 results to infer, obtaining similar results.

In conclusion, we confirmed the clinical significance of asthma clusters by longitudinally analyzing the data of our cohort. Our asthma clusters may not be immediately applied to clinical practice as further studies are needed to precisely characterize each cluster. An algorithm that can help allocate each new asthma patient into an appropriate cluster should be developedand it is hoped that cluster-specific pathogenesis could be revealed in the near future to aid the development of novel therapeutic strategies.

## Supporting Information

Figure S1 FEV_1_% predicted values during the 12-month follow-up period in each cluster after multiple imputations.
(DOCX)

Figure S2 ACT scores during the 12-month follow-up period in each cluster after multiple imputations.
(DOCX)

Figure S3 Percentage of use of systemic corticosteroids during the 12-month follow-up period in each cluster after multiple imputations.
(DOCX)

Figure S4 QLQAKA scores during the 12-month follow-up period in each cluster after multiple imputations.
(DOCX)

Table S1 FEV_1_% predicted values during the 12-month follow-up period in each cluster after multiple imputations.
(DOCX)

Table S2 ACT scores during the 12-month follow-up period in each cluster after multiple imputations.
(DOCX)

Table S3 Percentage use of systemic corticosteroids during the 12-month follow-up period in each cluster after multiple imputations.
(DOCX)

Table S4 QLQAKA scores during the 12-month follow-up period in each cluster after multiple imputations.
(DOCX)

Table S5 Characteristics of the four clusters of COREA patients.
(DOCX)
