# Supplementary material for: Clinical Significance of Asthma Clusters by Longitudinal Analysis in Korean Asthma Cohort
Source: PLoS One. 2013 Dec 31;8(12):e83540. doi: 10.1371/journal.pone.0083540 (PMC3877049; doi:10.1371/journal.pone.0083540)

**Figure S1. FEV_1_% predicted values during the 12-month follow-up period in each cluster after multiple imputations**


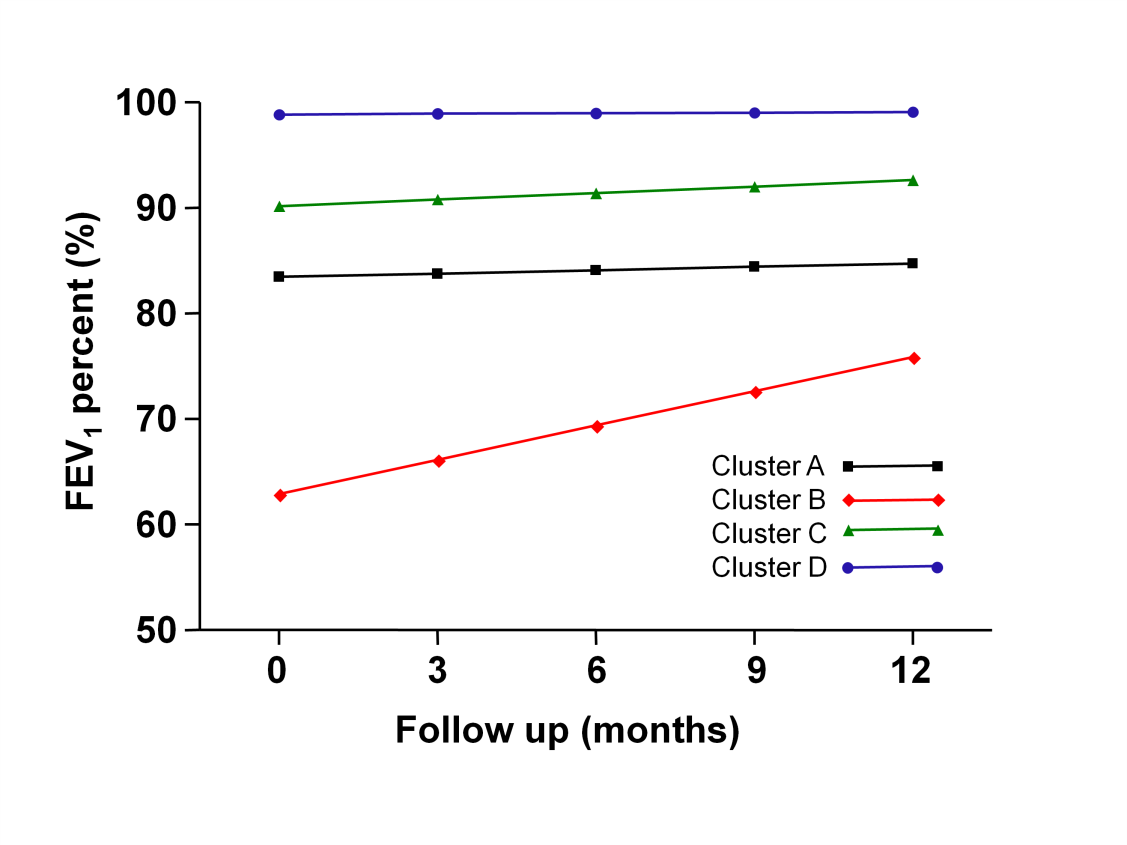

Supplement: Figure S1 — FEV1% predicted values during the 12-month follow-up period in each cluster after multiple imputations. (DOCX) [file pone.0083540.s001.docx]
