# Supplementary material for: Clinical Significance of Asthma Clusters by Longitudinal Analysis in Korean Asthma Cohort
Source: PLoS One. 2013 Dec 31;8(12):e83540. doi: 10.1371/journal.pone.0083540 (PMC3877049; doi:10.1371/journal.pone.0083540)
Supplement: Figure S4 — QLQAKA scores during the 12-month follow-up period in each cluster after multiple imputations. (DOCX) [file pone.0083540.s004.docx]

**Figure S4. QLQAKA scores during the 12-month follow-up period in each cluster after multiple imputations**

**
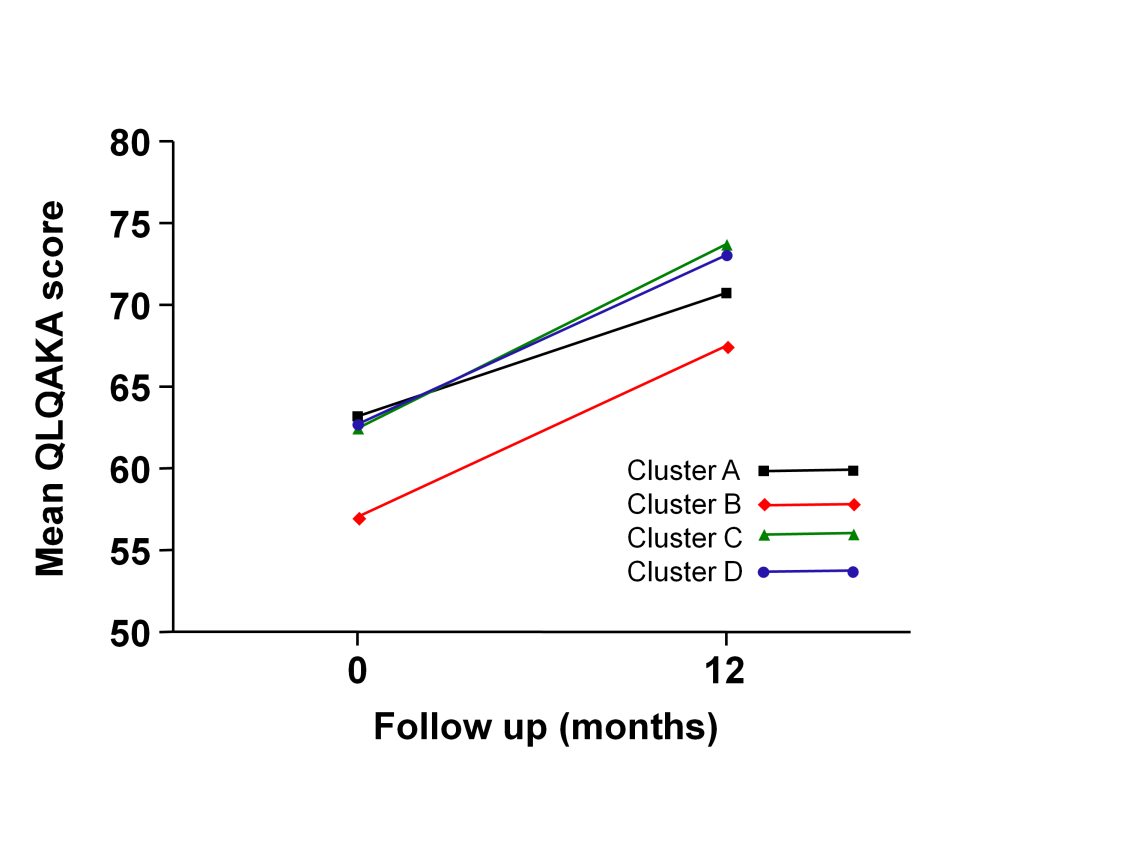
**
