# Supplementary material for: Clinical Significance of Asthma Clusters by Longitudinal Analysis in Korean Asthma Cohort
Source: PLoS One. 2013 Dec 31;8(12):e83540. doi: 10.1371/journal.pone.0083540 (PMC3877049; doi:10.1371/journal.pone.0083540)
Supplement: Table S1 — FEV1% predicted values during the 12-month follow-up period in each cluster after multiple imputations. (DOCX) [file pone.0083540.s005.docx]

**Table S1**. **FEV_1_% predicted values during the 12-month follow-up period in each cluster after multiple imputations**

|  | **A** | **B** | **C** | **D** |
| --- | --- | --- | --- | --- |
| **Months** | **Pred. Mean**  **(95% CI)** | **Pred. Mean**  **(95% CI)** | **Pred. Mean**  **(95% CI)** | **Pred. Mean**  **(95% CI)** |
| **0** | 83.47 (80.63–86.30) | 62.89 (60.88–64.91) | 90.18 (88.59–91.78) | 98.87 (97.27–100.47) |
| **3** | 83.78 (80.98–86.58) | 66.14 (64.14–68.14) | 90.80 (89.21–92.38) | 98.93 (97.30–100.56) |
| **6** | 84.10 (81.14–87.06) | 69.39 (67.26–71.51) | 91.41 (89.74–93.08) | 98.99 (97.13–100.84) |
| **9** | 84.42 (81.12–87.71) | 72.63 (70.27–74.99) | 92.02 (90.17–93.87) | 99.04 (96.82–101.27) |
| **12** | 84.73 (80.99–88.48) | 75.88 (73.19–78.56) | 92.63 (90.53–94.72) | 99.10 (96.43–101.77) |
