# Supplementary material for: Clinical Significance of Asthma Clusters by Longitudinal Analysis in Korean Asthma Cohort
Source: PLoS One. 2013 Dec 31;8(12):e83540. doi: 10.1371/journal.pone.0083540 (PMC3877049; doi:10.1371/journal.pone.0083540)
Supplement: Table S2 — ACT scores during the 12-month follow-up period in each cluster after multiple imputations. (DOCX) [file pone.0083540.s006.docx]

**Table S2. ACT scores during the 12-month follow-up period in each cluster after multiple imputations**

|  | **A** | **B** | **C** | **D** |
| --- | --- | --- | --- | --- |
| **Months** | **Pred. Mean**  **(95% CI)** | **Pred. Mean**  **(95% CI)** | **Pred. Mean**  **(95% CI)** | **Pred. Mean**  **(95% CI)** |
| **3** | 21.90 (20.92–22.87) | 21.16 (20.37–21.96) | 21.08 (20.50–21.66) | 22.07 (21.49–22.64) |
| **6** | 21.40 (20.32–22.47) | 20.81 (20.05–21.58) | 21.06 (20.33–21.80) | 22.18 (21.46–22.91) |
| **9** | 22.05 (21.08–23.02) | 20.87 (20.15–21.60) | 21.48 (20.84–22.12) | 22.30 (21.73–22.88) |
| **12** | 21.46 (20.48–22.43) | 21.02 (20.34–21.70) | 21.80 (21.20–22.40) | 22.26 (21.68–22.85) |
