# Supplementary material for: Clinical Significance of Asthma Clusters by Longitudinal Analysis in Korean Asthma Cohort
Source: PLoS One. 2013 Dec 31;8(12):e83540. doi: 10.1371/journal.pone.0083540 (PMC3877049; doi:10.1371/journal.pone.0083540)
Supplement: Table S3 — Percentage use of systemic corticosteroids during the 12-month follow-up period in each cluster after multiple imputations. (DOCX) [file pone.0083540.s007.docx]

**Table S3. Percentage use of systemic corticosteroids during the 12-month follow-up period in each cluster after multiple imputations**

|  | **A** | **B** | **C** | **D** |
| --- | --- | --- | --- | --- |
| **Months** | **Pred. Mean**  **(95% CI)** | **Pred. Mean**  **(95% CI)** | **Pred. Mean**  **(95% CI)** | **Pred. Mean**  **(95% CI)** |
| **3** | 0.173 (0.093–0.300) | 0.274 (0.189–0.379) | 0.123 (0.077–0.191) | 0.137 (0.087–0.210) |
| **6** | 0.260 (0.143–0.425) | 0.251 (0.162–0.366) | 0.146 (0.090–0.228) | 0.084 (0.039–0.170) |
| **9** | 0.195 (0.104–0.336) | 0.278 (0.189–0.390) | 0.157 (0.093–0.253) | 0.145 (0.084–0.239) |
| **12** | 0.287 (0.167–0.447) | 0.270 (0.180–0.384) | 0.078 (0.039–0.153) | 0.083 (0.043–0.153) |
