# Supplementary material for: Clinical Significance of Asthma Clusters by Longitudinal Analysis in Korean Asthma Cohort
Source: PLoS One. 2013 Dec 31;8(12):e83540. doi: 10.1371/journal.pone.0083540 (PMC3877049; doi:10.1371/journal.pone.0083540)
Supplement: Table S4 — QLQAKA scores during the 12-month follow-up period in each cluster after multiple imputations. (DOCX) [file pone.0083540.s008.docx]

**Table S4. QLQAKA scores during the 12-month follow-up period in each cluster after multiple imputations**

|  | **A** | **B** | **C** | **D** |
| --- | --- | --- | --- | --- |
| **Months** | **Pred. Mean**  **(95% CI)** | **Pred. Mean**  **(95% CI)** | **Pred. Mean**  **(95% CI)** | **Pred. Mean**  **(95% CI)** |
| **0** | 63.18 (57.62–68.73) | 57.03 (52.80–61.25) | 62.47 (58.87–66.06) | 62.70 (58.86–66.54) |
| **12** | 70.74 (65.56–75.93) | 67.51 (62.75–72.27) | 73.71 (70.23–77.20) | 73.05 (69.00–77.10) |
