# Supplementary material for: Clinical Significance of Asthma Clusters by Longitudinal Analysis in Korean Asthma Cohort
Source: PLoS One. 2013 Dec 31;8(12):e83540. doi: 10.1371/journal.pone.0083540 (PMC3877049; doi:10.1371/journal.pone.0083540)
Supplement: Table S5 — Characteristics of the four clusters of COREA patients. (DOCX) [file pone.0083540.s009.docx]

**Table S5. Characteristics of the four clusters of COREA patients.**

|  | **Cluster A**  **(n=81)** | **Cluster B**  **(n=151)** | **Cluster C**  **(n=253)** | **Cluster D**  **(n=239)** | **P value** |
| --- | --- | --- | --- | --- | --- |
| **Gender (% of male)** | **97.4** | **41.3** | **47.2** | **25.8** | **<0.001** |
| **Age at onset, yr** | **46.2±12.6** | **38.8±15.1** | **21.8±9.4** | **47.8±9.7** | **<0.001** |
| **Body mass index (kg/m^2^)** | **24.71±2.43** | **23.50±3.09** | **23.09±3.45** | **24.35 ± 3.07** | **<0.001** |
| **Smoking (pack-years)** | **34.4±14.1** | **3.2±6.5** | **2.0±3.7‡** | **2.0± 5.3** | **<0.001** |
| **Atopy, % positive** | **34.6** | **49.7** | **66.0** | **53.6** | **<0.001** |
| **History of HU (% positive)** | **22.2** | **46.4** | **36.8** | **23.4** | **<0.001** |
| **Rhinitis (% of positivity)** | **58.3** | **65.3** | **73.4** | **65.0** | **0.056** |
| **PostBD FEV1 (%)** | **82.47±16.45** | **56.48±13.7** | **88.62±11.24** | **97.94±11.5** | **<0.001** |
| **PostBD % increased of FEV1** | **5.90±7.75** | **12.00±12.07** | **6.24±7.52** | **4.34±6.56** | **<0.001** |
| **PostBD FEV1/FVC (%)** | **71.44±11.76** | **67.84±12.40** | **84.76±8.69** | **80.25±7.40** | **<0.001** |
| **PC20 (mg/ml)** | **4.95±6.14** | **3.11±5.35** | **5.49±6.04** | **5.64±6.47** | **0.03** |
| **Blood eosinophils (%)** | **4.80± 4.33** | **5.60± 5.48** | **5.50± 6.43** | **5.02± 4.70** | **0.612** |
| **Blood eosinophil count (/mm^3^)** | **388.4±376.6** | **409.6±454.2** | **377.9±354.3** | **331.0±301.1** | **0.247** |
| **Log blood eosinophil count** | **2.42±0.44** | **2.40±0.47** | **2.41±0.41** | **2.34±0.42** | **0.445** |
| **Blood neutrophil count (/mm^3^)** | **4397± 1971** | **4672± 3168** | **4107± 1775** | **3631± 1606** | **<0.001** |
| **Serum CRP (mg/dl)** | **0.78± 2.43** | **0.61± 0.90** | **0.21± 0.35** | **0.37± 0.96** | **0.02** |
| **Serum uric acid levels (mg/dl)** | **5.85± 1.35** | **5.13± 1.51** | **5.10± 1.39** | **4.85± 1.60** | **<0.001** |
| **Serum Total IgE (Log IU/L)** | **2.41±0.62** | **2.26±0.59** | **2.35±0.60** | **2.14± 0.61** | **0.008** |

**Comparison between study groups is based on One-way ANOVA analysis for continuous variables and a χ^2^ test for proportions. Data indicate means ± SDs. Adapted from Eur Respir J, 2013;41:1308-1314.**
